# Thermophysical Measurements in Liquid Alloys and Phase Diagram Studies

**DOI:** 10.3390/ma12233999

**Published:** 2019-12-02

**Authors:** Yuri Kirshon, Shir Ben Shalom, Moran Emuna, Yaron Greenberg, Joonho Lee, Guy Makov, Eyal Yahel

**Affiliations:** 1 Department of Materials Engineering, Ben-Gurion University of the Negev, Beer Sheva 84105, Israel; iurik@post.bgu.ac.il (Y.K.); shirbe@bgu.ac.il (S.B.S.); 2 Physics Department, Nuclear Research Centre Negev, Beer Sheva 84190, Israel; morankm131@gmail.com (M.E.); yaron300@gmail.com (Y.G.); eyalyahel@gmail.com (E.Y.); 3 Department of Materials Science and Engineering, Korea University, Seoul 02841, Korea; joonholee@korea.ac.kr

**Keywords:** thermal analysis, sound velocity, electrical resistivity, density, liquid metals, calculation of phase diagrams (CALPHAD)

## Abstract

Towards the construction of pressure-dependent phase diagrams of binary alloy systems, both thermophysical measurements and thermodynamic modeling are employed. High-accuracy measurements of sound velocity, density, and electrical resistivity were performed for selected metallic elements from columns III to V and their alloys in the liquid phase. Sound velocity measurements were made using ultrasonic techniques, density measurements using the gamma radiation attenuation method, and electrical resistivity measurements were performed using the four probe method. Sound velocity and density data, measured at ambient pressure, were incorporated into a thermodynamic model to calculate the pressure dependence of binary phase diagrams. Electrical resistivity measurements were performed on binary systems to study phase separation and identify phase transitions in the liquid state.

## 1. Introduction

Phase diagrams are employed extensively in material science and associated technological applications in industry. For decades, phase diagrams at ambient pressure have been explored by measuring thermophysical quantities and by theoretical modeling. Phase boundaries can be determined directly by experimental techniques such as thermal analysis, including using differential thermal analysis (DTA) or differential scanning calorimetry (DSC), volume changes (e.g., dilatometry), electrical resistivity measurements, or X-ray diffraction (XRD) to identify structural changes [1]. 

Experimental methods are costly when mapping a phase diagram of binary and ternary systems over a wide range of compositions and temperatures, due to the large number of experiments required. Hence, direct measurements of phase transitions are often complemented by thermodynamic modeling of phase diagrams, typically determined by the Gibbs free energy, which describes the lowest energy, most stable phases, as a function of the thermodynamic parameters, temperature, composition and occasionally pressure. Calculation of phase diagrams (CALPHAD) methodology is commonly used to model phase diagrams. It is based on thermodynamic considerations and empirical databases, extrapolating the thermodynamic parameters to calculate these complex systems [2,3]. Several software packages have been developed (e.g., ChemSage, WinPhad, PANDAT, and ThermoCalc) that employ the CALPHAD methodology to calculate the phase diagrams of complex systems at ambient pressure [4]. A major challenge of this approach is the need for reliable thermodynamic property databases to calculate the phase diagram. Thus, extensive thermodynamic measurements of enthalpy, heat capacity. and activity (through electromotive force (EMF) measurements and vapor pressure studies) have been conducted and analyzed [5] to support this venture. 

Pressure can affect physical properties and, in many cases, stabilize new phases. Physical properties of alloys under high pressure are at the focus of planetary research, as well as having advanced technological applications [6,7,8]. The emergence of high pressure technologies enables the scientific community to explore regions of phase diagrams at elevated pressures and high temperatures, which were inaccessible in the past [9,10]. However, numerous experimental challenges remain. The versatile and popular high-pressure apparatus of the diamond anvil cell (DAC) is widely used to measure structural changes under high pressure. Currently, measurements can be performed at a pressure range of 0–500 GPa and at temperatures ranging from cryogenic to thousands of degrees. At a lower pressure range one can find other techniques such as piston cylinder, multi-anvil press, and the Paris–Edinburgh large volume cell. To date, phase diagrams of elements under pressure are relatively well-known and established [11] but the database for binary systems is limited. For ternary systems, the database is practically nonexistent. 

To establish phase diagrams in the liquid state, additional experimental challenges are presented, for example, chemical reactivity of the liquid at high temperatures with structural materials and effects associated with high vapor pressure. Hence, information on the properties of liquids at high temperatures, and even more so at high pressures, is limited. The scarce data that exists has relatively large experimental error, and as a result, there are discrepancies between reported thermophysical values. Even for elemental systems there are open questions, for example, the shape of the melting curve [12] and the possible existence of liquid–liquid transitions [13]. Direct measurements of the changes in the liquid structure and their interpretation can be challenging [14]. Indirect studies of phase transformations in the liquid using classical thermal analysis approaches are rare.

The study of liquid binary systems requires even greater experimental effort, due to the need to carefully measure a range of compositions and include phase separation phenomena. Therefore, this area of research remains relatively unexplored. Recently, we have developed a methodology to determine the pressure and temperature dependence of the excess interaction parameters in liquid solution, from ambient pressure measurements of density, heat capacity, and thermal expansion. This approach enables the construction of binary alloy phase diagrams at low pressures of up to several GPa [15,16].

The purpose of this paper is to provide an overview of thermophysical measurements and associated models that support the construction of phase diagrams in liquid binary alloys of columns III to V and their constituent elemental systems. These systems exhibit complex phase diagrams under pressure and their thermophysical properties are often anomalous. Their low melting points make them easily accessible for experimental study, and thus they are ideal candidates for developing new approaches to phase diagram studies. In particular, we have developed techniques to measure the density and sound velocity to high accuracy in liquid metals, which is required to establish reliable diagrams. The results of such measurements have been incorporated into a model, describing the free energy of the liquid alloys and predicting the pressure dependence of binary phase diagrams. Electrical measurements provide a complementary technique to explore phase transitions and the limits of liquid phase stability. Such measurements have been applied at both ambient and high pressures to identify phase stability limits. This set of techniques and accurate measurements provides new pathways to study the liquid phase diagram under pressure.

## 2. Density 

Density is a fundamental thermodynamic property of matter, relevant to the determination of other physical parameters, such as viscosity, surface tension, heat capacity, and even to the description of the radial density function (RDF) from which the short-range order can be evaluated. In the lieterature, several experimental techniques have been described for measuring the density of liquid metals. These include the maximum bubble pressure, Archimedean, liquid drop, dilatometric, and gamma radiation attenuation methods [17]. In our research we implemented a high-accuracy measurement based on gamma radiation attenuation.

### 2.1. Gamma Radiation Attenuation Method

This method is based on measuring the attenuation of gamma radiation passing through a sample. A γ radiation source is located on one side of the furnace, containing the sample, and a γ radiation detector is placed on the other side. The beam, passing through the liquid sample, is attenuated, and the density can be calculated by:

(1) $$I=I_{0}e^{\left(-\mu \rho x\right)}$$

where I_0_ and I are the intensity of the beam before the sample and the intensity measured at the detector, respectively; μ, ρ, and x are the absorption coefficient, the density, and the optical thickness of the entire path from the source to the detector. The contribution of the liquid can be deduced from premeasuring the transmission in the exact experimental configuration without the sample. To eliminate the thermal expansion of the experimental setup, we measured relative density. This method is mostly used for materials with high density, in which the attenuation due to the liquid is significant as compared with the structural materials, to obtain a large signal-to-noise ratio.

The experimental error is due to the following two main factors: (i) statistical error of the detector counts and (ii) the radiation background intensity. The first is assumed to have Lorentz distribution, and hence follows ΔI = √I. To reduce the statistical error, we significantly increased the measurement time. To reduce background radiation we subtracted reference measurements of the radiation intensity measured without a sample as a function of temperature, with the same temperature profile. As a result, the relative error of the density is proportional to the squared sum of the relative errors of the intensities with and without the liquid sample. 

### 2.2. Density Results

The density of pure lead and bismuth was measured by the gamma radiation attenuation method. The radiation source used was ^137^Cs with a characteristic energy of 662 Kev. This source is monoenergetic, and has a relatively long half-life and a large cross-section for absorption in the liquid samples. The crucible was made of fused quartz, which has low thermal expansion, with a square cross-section for simple geometry. The detector chosen was a CsI scintillator due to its high sensitivity and dynamic range. 

**Figure 1:**
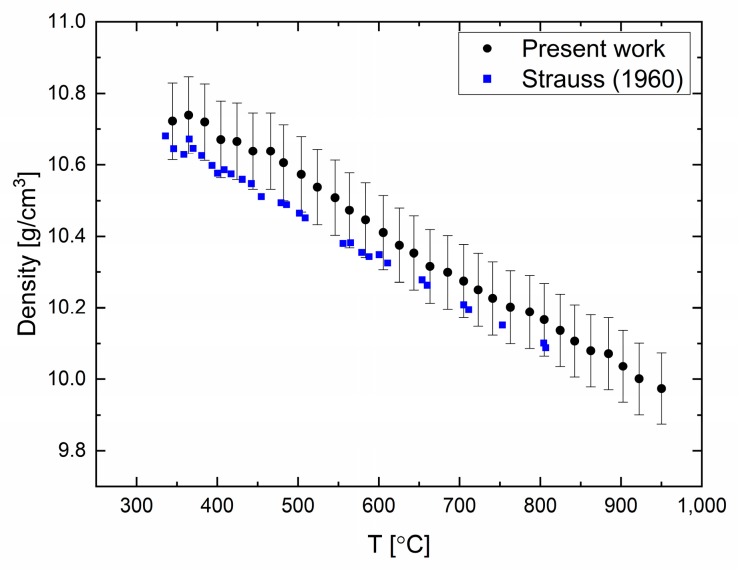
Lead density as a function of temperature compared with data from reference [18].

Density measurements in elemental lead (99.999% purity) were carried out in the temperature range of 330 to 950 °C every 20 °C, as shown in Figure 1. It can be seen that the density decreases monotonically as the temperature is increased, without discontinuities or changes in slope. The uncertainty (standard deviation) in these measurements was estimated to be 1% due to the sampling statistics. The thermal expansion of liquid Pb can be evaluated, and was found to be 1.22 × 10^−4^ K^−1^. Comparing the present data with previous measurements [18] of the density obtained by the Archimedean method shows a small difference that is contained by the experimental error. The difference in the density at melting point was found to be less than 1%. 

**Figure 2:**
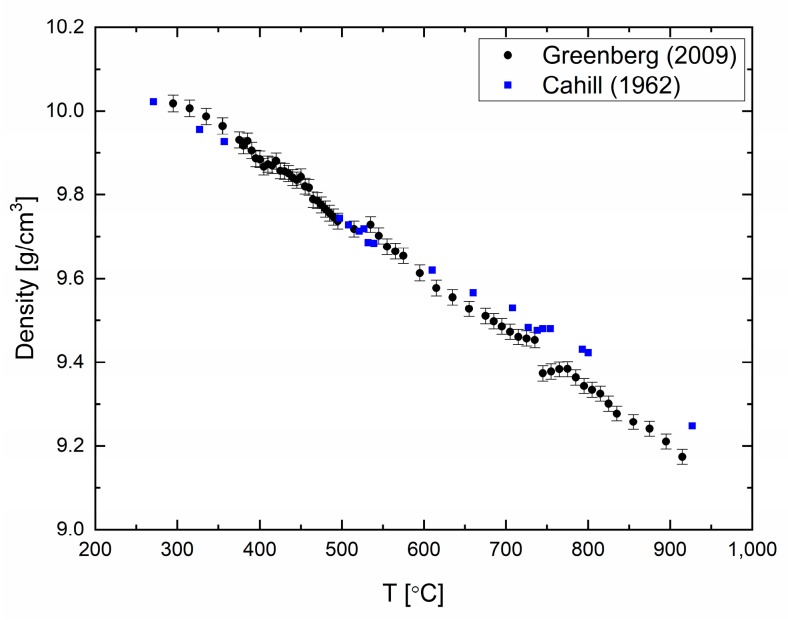
Bismuth density as a function of temperature reproduced with permission from Ref. [19] compared with data from Ref. [20].

The density of elemental bismuth (99.999% purity) was measured and reported in [19] in a temperature range of 200–1000 °C every 2 °C to 5 °C (Figure 2). A good agreement between the previously published data and our measurements can be seen in Figure 2. Similar to liquid Pb, liquid Bi expands as the temperature increases. The errors in the measurements are calculated to be 0.1%. Density changes are observed at ca. 550 °C and 720 °C, which might point to structural changes in the liquid phase of Bi [19]. 

## 3. Sound Velocity

**Figure 3:**
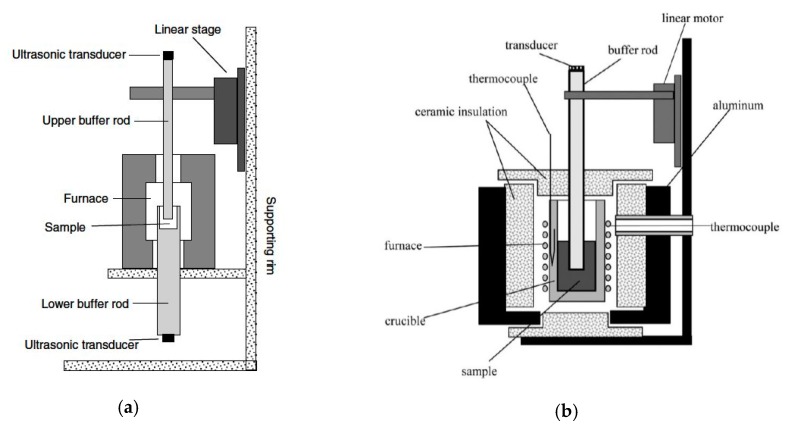
Schematic view of (**a**) the ultrasonic pulse transmission experimental measurement apparatus reproduced with permission from [23] and (**b**) the ultrasonic pulse-echo experimental measurement apparatus reproduced with permission from [24].

Sound velocity is an important thermodynamic quantity, sensitive to changes in the material properties. To measure the velocity of sound in liquids, ultrasonic or laser methods can be employed [21,22]. In optical systems, stress waves are generated, which result in surface motion. This method is suitable for measuring sound velocity at very high temperatures and pressures. The two systems used in our studies are modifications of the well-known ultrasonic method designed for high-accuracy measurement. The first is based on transmission and includes two transducers to measure the transmitted wave, one to generate the acoustic wave and the other to receive it (i.e., the wave travels only once in the liquid sample). The second apparatus is based on reflection of the emitted wave from the base of the sample container back to the transducer. Both these tabletop systems are presented schematically in Figure 3 and operate at ambient pressure and have a unique high accuracy that is preserved at elevated temperatures. 

### 3.1. Sound Velocity Measurements by Ultrasonic Techniques

The pulse transmission system is composed of two ceramic buffer rods that serve as waveguides and two electronic ultrasound transducers that are attached to them, the first transmitting an acoustic wave and the second receiving it. The buffer rods allow the transducers to operate below the maximum working temperature. The crucible containing the liquid sample is machined at the top of the lower buffer rod. An ultrasonic elastic wave travels through the upper buffer rod, which is immersed in the liquid metal, traveling through the liquid sample and continuing to the lower buffer rod. The upper rod is attached to an accurate linear motor. By moving this rod a known distance of Δx, the traveling time of the sound wave is increased in Δt, and the sound velocity is deduced from the ratio C = Δx/Δt (i.e., it is a differential measurement). The uncertainty of the measurement estimated to be 0.1% to 0.35%, mainly due to the finite precision of the linear motor. More details on this measurement method are in reference [23]. 

The pulse-echo ultrasonic technique is, in principle, similar to the pulse transmission method. However, in this method we used a single buffer rod and a transducer that operates as both transmitter and receiver of the acoustic wave. The liquid sample is held in a ceramic crucible, the bottom of which serves as a reflector. To eliminate uncertainty as to the distance the ultrasonic wave travels through the liquid, the rod is translated by a known distance, Δx. As the wave travels a distance of 2Δx in the liquid metal with extra time Δt, the sound velocity is determined by c = 2Δx/Δt. This method has a similar error, as the limited accuracy of the linear motor is the same, and it is estimated to be 0.2%. More details on this measurement technique are in reference [24]. 

In both cases, the experimental apparatus was placed in a glovebox with a protective gaseous atmosphere of high-purity argon in a constant flow mode. 

The transmission technique benefits from a higher signal-to-noise ratio, due to the short distance the sound wave travels through the liquid, and due to the attenuated shear waves in the thicker lower rod. The pulse-echo technique is easier to apply and has a simple setup, however, its main drawback is that the bottom of the crucible needs to be polished to a high quality to minimize losses upon reflection of the sound wave. In addition, the amplitude of the wave is more attenuated since the sound wave travels through the liquid twice.

### 3.2. Sound Velocity Results

#### 3.2.1. Elemental Systems

**Figure 4:**
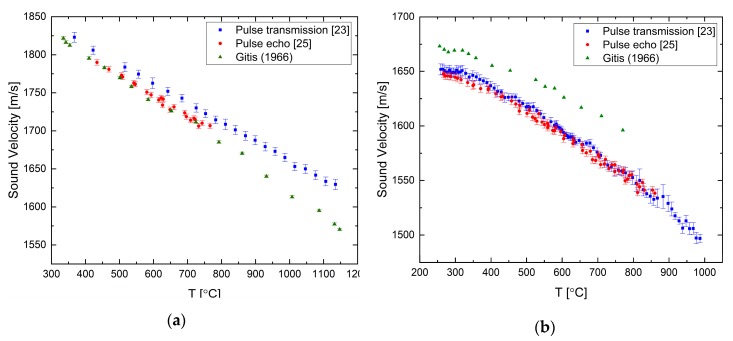
The temperature dependence of the sound velocity in (**a**) pure liquid lead [23,25] compared with data from [26] and in (**b**) pure liquid bismuth [23,25] compared with data from [26].

The sound velocity in pure liquid lead and bismuth has been measured by both of the ultrasonic techniques presented in the previous section as reported in [23,25]. For liquid Pb, the sound velocity decreases as the temperature is increased with a constant rate, as shown in Figure 4a. The difference between the measured and literature data increases with temperature and reaches about 1.5% at ca. 1000 °C. Our two methods agree reasonably well within the overlapping measurement range. For the liquid Bi (Figure 4b), the overall tendency is a negative temperature coefficient, but it shows a more complex behavior, namely, that the temperature coefficient changes with temperature. There is a good agreement between the two techniques. 

**Figure 5:**
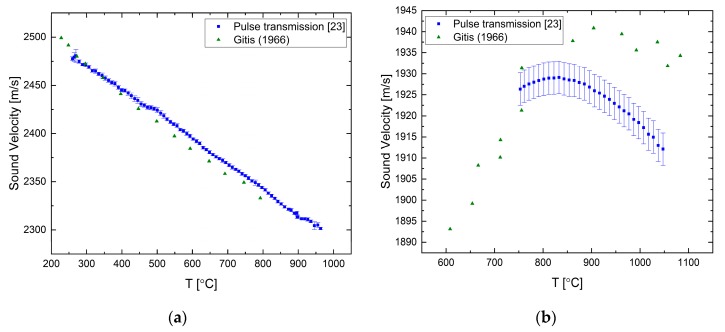
The temperature dependence of the sound velocity in (**a**) pure liquid tin [23] compared with data from [26] and in (**b**) pure liquid antimony [23] compared with data from [26].

The velocity of sound was measured for elemental tin and antimony in [23] using the pulse transmission method only. For tin, we observe a normal sound velocity dependency, with an excellent agreement with previous data, as shown in Figure 5a. Antimony has an anomalous behavior as presented in Figure 5b. Up to a temperature of ~830 °C the sound velocity increases with increasing temperature, up to a maximum, then, decreasing nonlinearly.

**Figure 6:**
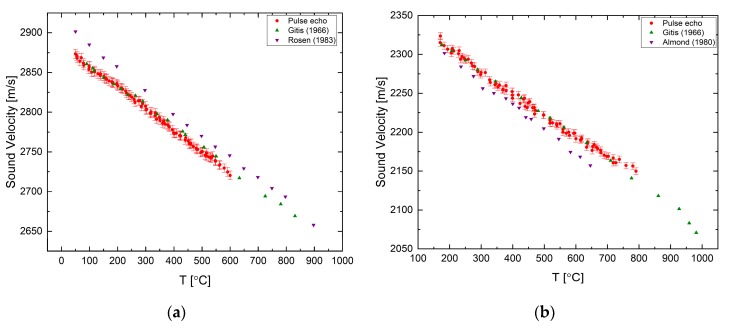
The temperature dependence of the sound velocity measured in (**a**) pure liquid gallium compared with data from [27,28] and in (**b**) pure liquid indium compared with data from [27,29].

The sound velocity of liquid gallium (99.99% purity) and liquid indium (99.999% purity) were measured using the pulse-echo setup and the results are presented in Figure 6 and detailed in the Supplementary Material. Both elements display normal behavior and a good agreement between the measured values and previously published data.

#### 3.2.2. Binary Systems

**Figure 7:**
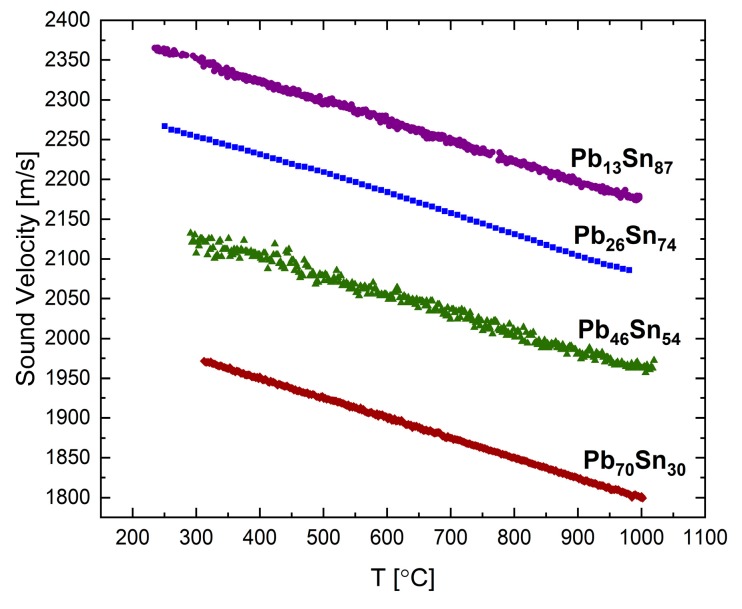
The temperature dependence of the sound velocity in the liquid Pb-Sn system at different alloy compositions adapted with permission from [15].

The sound velocities in liquid Pb-Sn and Bi-Sn were measured in [15] using the transmission method. Figure 7 presents the sound velocity as a function of temperature for the following four compositions of the Pb-Sn system up to 1000 °C: Pb_13_Sn_87_, Pb_26_Sn_74_, Pb_46_Sn_54_, and Pb_70_Sn_30_ (at.%). For all the compositions a normal behavior is observed, as in the two component elements.

**Figure 8:**
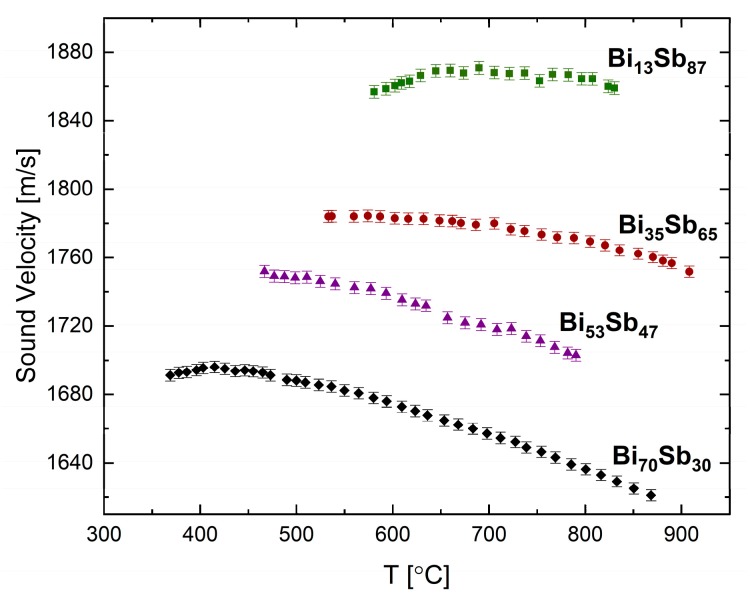
Sound velocity in the liquid Bi-Sb system as a function of temperature at selected alloy compositions, adapted with permission from [24].

The sound velocity of binary systems Bi-Pb [25] and Bi-Sb [24] were measured by the pulse-echo technique. In Figure 8, we present some results of the Bi-Sb isomorphous binary alloy. Measurements in the liquid phase were carried up to temperatures of ca. 900 °C for the following selected compositions: Bi_13_Sb_87_, Bi_35_Sb_65_, Bi_53_Sb_47_, and Bi_70_Sb_30_ (all in at.%). In this system, the Sb-rich alloys, Bi_13_Sb_87_ and Bi_35_Sb_65_, display anomalous behavior similar to Sb, but with less significant trends. The temperature at which the sound velocity is maximal decreases from ~830 °C to ~700 °C at 13% Bi and ~520 °C at 35% Bi alloy composition. As the Bi concentration is increased the temperature dependence of the sound velocity becomes more linear, and for the Bi-rich alloy, Bi_70_Sb_30_, a semi-normal behavior at the low temperatures near the solidification is observed, similar to Bi.

## 4. Modeling Binary Phase Diagrams under Pressure

Phase diagrams of binary alloys are expected to vary with pressure. Measuring thermophysical properties under pressure is experimentally challenging in addition to the vast amount of data required to construct the diagram as a function of temperature, composition, and pressure. Therefore, we proposed to follow a different route, i.e., to calculate the pressure-dependent phase diagram with input from ambient measurements of sound velocity and density to obtain the variation of interaction parameters with pressure. Lastly, information on the temperature-pressure phase diagram of the elements constituting the binary system is required. 

The equilibrium condition to determine phase line is an equality of the chemical potentials of the same component in the two different phases, calculated from the Gibbs free energy:

(2) $$\mu_{i}={\left(\frac{\partial G}{\partial N_{i}}\right)}_{P,T}$$

where µ_i_ is the chemical potential of component i, G is the Gibbs free energy, and N_i_ is the number of particles. 

Most binary alloy systems do not behave as ideal solutions. In systems with an asymmetric miscibility gap, the Gibbs free energy can be expressed to the lowest order in composition in the form of a sub-regular solution:

(3) $$G=X_{A}G_{A}+X_{B}G_{B}+\mathrm{RT}\left(X_{A}{\mathrm{lnX}}_{A}+X_{B}{\mathrm{lnX}}_{B}\right)+J_{0}X_{A}X_{B}+J_{1}X_{A}X_{B}\left(X_{A}-X_{B}\right)$$

where X_A_ and X_B_ are the atomic fractions of each component, G_A_ and G_B_ are the partial Gibbs energy, and J_0_ and J_1_ are the regular and sub-regular interaction coefficients. The latter depend on pressure, and the variation of those parameters with pressure is a crucial input for the calculated phase diagram under pressure.

The pressure dependence of the interaction coefficient may be expanded to the second order and the deviation of the molar volume from its ideal value can be expressed in the following manner:

(4) $$\delta V=\frac{\partial J_{0}}{\partial P}X_{A}X_{B}+\frac{\partial J_{1}}{\partial P}X_{A}X_{B}\left(X_{A}-X_{B}\right)$$

(5) $$\frac{\partial \delta V}{\partial P}=\frac{\partial^{2}J_{0}}{\partial P^{2}}X_{A}X_{B}+\frac{\partial^{2}J_{1}}{\partial P^{2}}X_{A}X_{B}\left(X_{A}-X_{B}\right)$$

The sound velocity of the liquid at ambient pressure (C_s_), is related to the molar volume and the adiabatic compressibility (K_S_) by:

(6) $$\frac{1}{C_{s}{}^{2}}={\rho K}_{s}=-\frac{M}{V^{2}}{\left(\frac{\partial V}{\partial P}\right)}_{S}$$

Hence, the sound velocity of an ideal solution, which is the velocity of the elements weighted by the relative composition, can be related to the measured one using Equation (6) to obtain the relation:

(7) $${\left(\frac{C_{\mathrm{id}}}{C_{s}}\right)}^{2}-1=-\frac{2\delta V}{V_{\mathrm{id}}}+\frac{\frac{\partial \delta V}{\partial P}}{X_{A}\frac{1}{C_{s,A}{}^{2}}\left(-\frac{V_{A}{}^{2}}{M_{A}}\right)+X_{B}\frac{1}{C_{s,B}{}^{2}}\left(-\frac{V_{B}{}^{2}}{M_{B}}\right)}+0\left({\delta V}^{2}\right)$$

The pressure dependence of the interaction parameter, J(P), is derived from measurements of the sound velocity and density performed at ambient pressure to determine the deviation of the molar volume from its ideal values (δV) to estimate $\frac{\partial \delta V}{\partial P}$. Extending the CALPHAD methodology, we calculated the phase diagrams of several binary alloys under pressure, including both isomorphous and eutectic systems which included: Bi-Sb, Bi-Sn, Pb-Sn [15], and Bi-Pb [25]. The model cannot represent the formation of new high-pressure phases in the P-T diagram of the alloy. The limitation on the pressure range arises from the fact that the interaction parameters are expanded only up to the second order.

**Figure 9:**
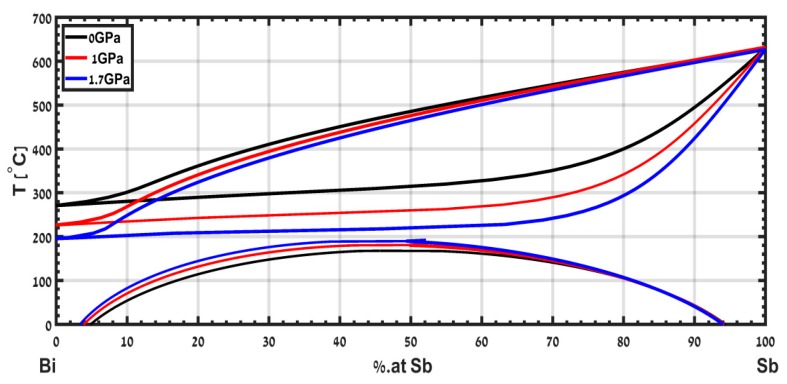
Calculated phase diagram of the isomorphic system Bi-Sb from ambient pressure up to pressure of 1.7 GPa adapted with permission from [15].

The phase diagram of the isomorphous binary system Bi-Sb was calculated in [15] up to a pressure of 1.7 GPa and is shown in Figure 9. The solidus decreases significantly with pressure, while the liquidus slightly decreases, mainly due to the anomaly in the melting temperature of the Bi with respect to pressure. The calculated phase diagram of this alloy is limited to a pressure of 1.7 GPa. Extension of this study to higher pressures is a subject for future study.

**Figure 10:**
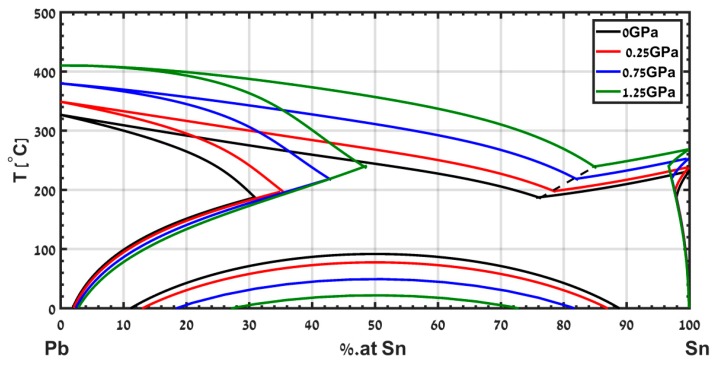
Calculated phase diagram of the eutectic system Pb-Sn from ambient pressure up to pressure of 1.25 GPa, adapted with permission from [15].

A different example is the eutectic phase diagram of Pb-Sn, which has been calculated in [15] up to a pressure of 1.25 GPa and is presented in Figure 10. The model captures the shifts in the eutectic composition and temperature. This useful information is hard to obtain experimentally. Note that the eutectic point shifts to a composition rich in Sn, and the eutectic temperature increases with increasing pressure. 

## 5. Electrical Resistivity

Electrical resistivity is one of the basic transport properties of a material. It is a useful experimental tool for studying phase transformations in solid and in liquid phases, for example, to identify melting point due to the abrupt change in resistivity resulting from the loss of the long-range order. In the literature, two classes of experimental techniques for resistivity measurements in liquid metals have been proposed, i.e., non-contact and contact methods. In this study, we applied a contact method implemented using a tabletop setup that was designed and built to be simple, modular, and accurate. 

**Figure 11:**
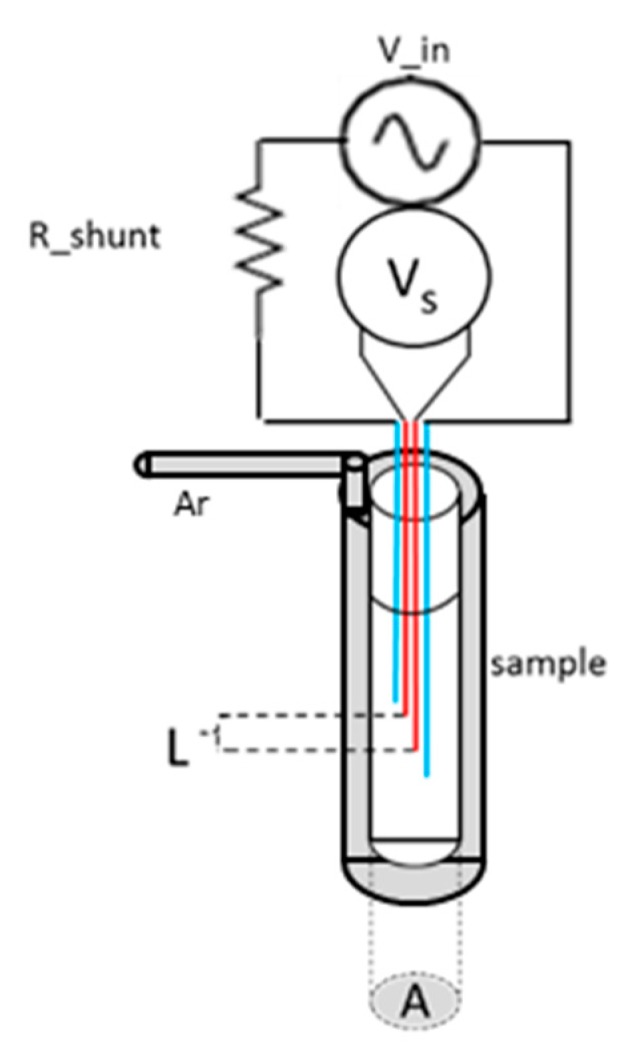
Schematic of the measuring system.

The experimental apparatus is based on an alternating current (AC) source with the four-point probe technique commonly used in the literature. The use of AC instead of direct current (DC) reduces the Seebeck effect and, by using a known reference frequency, enables better elimination of external noise [30]. In the present setup, the melt is held in a quartz test tube and is in direct contact with the immersed electrodes. Measurements are carried out under a protective gaseous argon atmosphere in a constant flow mode to avoid the enhanced reactivity of liquid metals at high temperature with the structural materials constructing the experimental chamber, which were chosen to have low reactivity. Figure 11 displays a schematic view of the apparatus. 

An alternating current is supplied to the sample, and the voltage drop across the sample is measured. The calculated resistivity (ρ_sample_) from the measured voltage drop is as follows:

(8) $$\rho_{\mathrm{sample}}=V_{\mathrm{meas}}\frac{R_{\mathrm{shunt}}}{V_{\mathrm{in}}}G$$

where G is the geometric constant of the cell, V_meas_ the measured voltage, V_in_ the input voltage, and R_shunt_ the shunt resistor. The precise cell dimensions are needed to convert the measured voltage to resistivity. However, if only the relative resistivity or the temperature coefficient is of interest, one can ignore the geometric constant. 

The estimated error consists of statistical and systematic errors. The main contribution to systematic error arises from cell dimensions. The measured voltage is averaged over a temperature window of 5 ºC, and the standard deviation of the statistical distribution of the voltage within this window is calculated. The error is, therefore, presented in Equation (9).

(9) $$\frac{\Delta \rho }{\rho }=\sqrt{\left(2{\left(\frac{\Delta V_{meas}}{V_{meas}}\right)}^{2}+{\left(\frac{\Delta R_{shunt}}{R_{shunt}}\right)}^{2}+{\left(\frac{\Delta L_{elec}}{L_{elec}}\right)}^{2}+{\left(\frac{\Delta A}{A}\right)}^{2}\right)}$$

where A and L are the sample’s cross-section and length, determined by the voltage electrodes. We assume that only the voltage measurement contains both statistical and systematic errors. Other terms consist of systematic errors only. 

The error in determining the absolute value of electrical resistivity is approximately 3%, a major part of which is derived from the uncertainty of the geometric dimensions of the cell. Consequently, the error in determining the relative values of the resistivity is only 0.1%.

### 5.1. Electrical Resistivity Results

#### 5.1.1. Elemental Systems 

**Figure 12:**
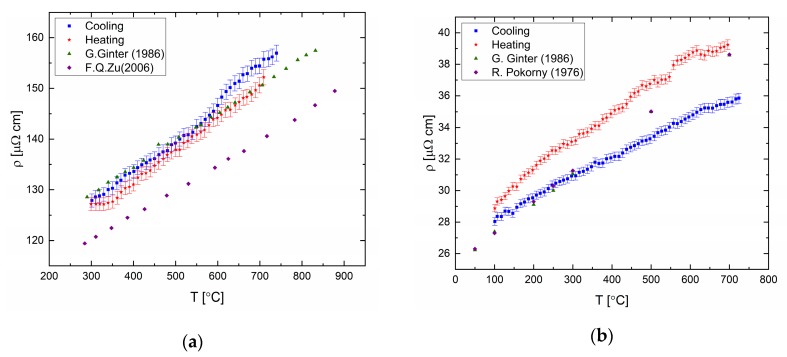

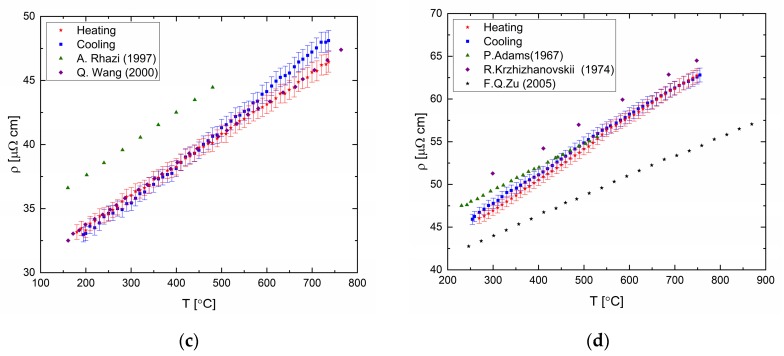
Resistivity of pure metals (**a**) bismuth and references [31,32]; (**b**) gallium and references [31,33]; (**c**) indium and references [34,35]; and (**d**) tin and references [36,37,38]. Cooling and heating rate of 60 °C/h.

The electrical resistivity was measured for elemental bismuth, tin, indium (99.999% purity), and gallium (99.99% purity). The results are presented in Figure 12 and in tabular form in the Supplementary Material. The resistance varies linearly with temperature in the liquid state, upon heating and cooling cycles. Deviation of the resistivity-temperature coefficient from published data can be seen for the temperature coefficient upon heating and cooling cycles for Bi and Ga.

#### 5.1.2. Binary Systems 

The electrical resistance of Bi-Ga and Ga-In alloys (prepared from the same sources as the elemental samples above) was measured as a function of temperature and composition (see Supplementary Material). To ensure reliable data, the resistivity of every composition was measured for two different samples, each undergoing at least three cooling and heating cycles.

**Figure 13:**
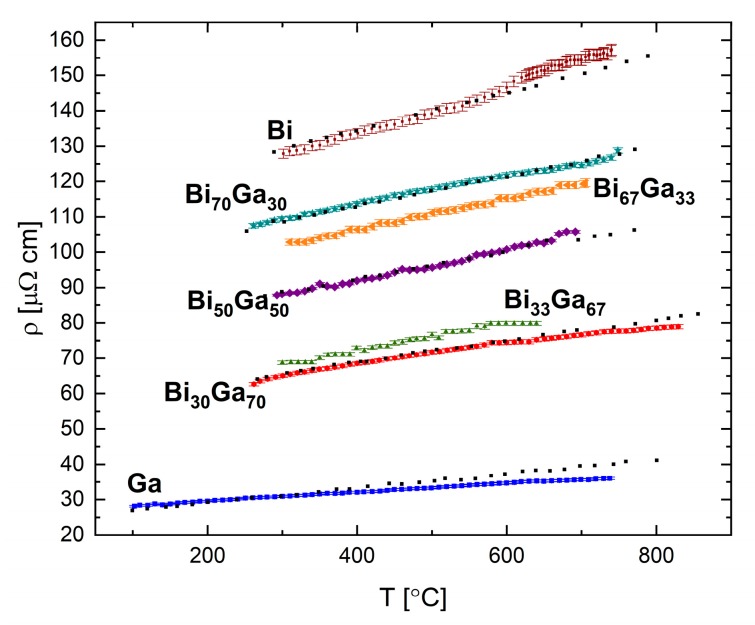
Electrical resistivity in the liquid Bi-Ga system as a function of temperature at selected alloy compositions. The uncertainty is smaller than the symbol size. The black dots are data from [31].

The resistivity of the Bi-Ga system was measured for the following compositions: Bi_30_Ga_70_, Bi_33_Ga_67_, Bi_50_Ga_50_, Bi_67_Ga_33_, and Bi_70_Ga_70_ (in at.%) and the results are presented in Figure 13. A linear trend is found for all measured compositions, in which the resistivity increases with increasing temperature. 

**Figure 14:**
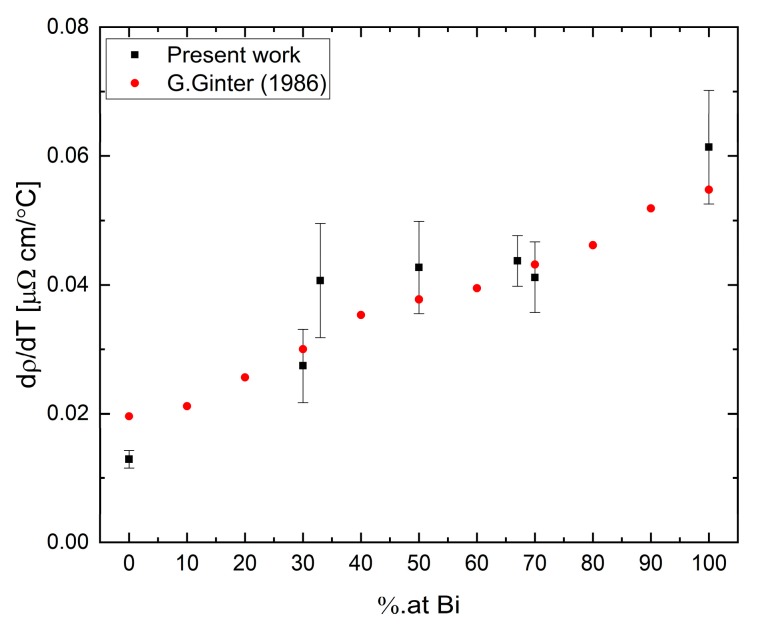
Resistivity coefficient of Bi-Ga alloy vs. Bi concentration.

The temperature coefficients $\frac{d\rho }{dT}$ were calculated by a linear fit to the resistivity data and are presented as a function of Bi concentration in Figure 14. The coefficient values are calculated over the measured temperature range displayed in Figure 13. Our results suggest a possible correlation of the temperature coefficient in the Bi-Ga system to a second-order polynomial as a function of composition. Parabolic dependence is an expected behavior for alloys containing metals with mixed valences [39]. 

**Figure 15:**
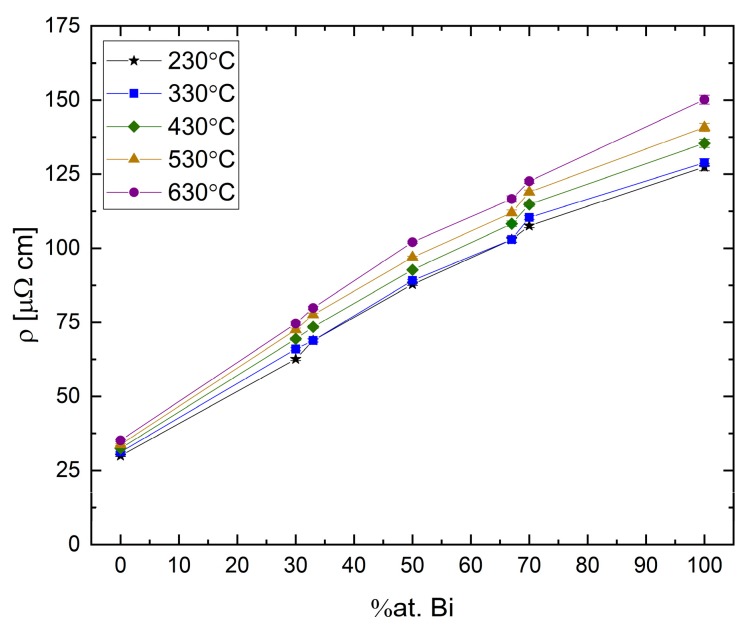
Absolute resistivity of Bi-Ga binary alloy vs. Bi concentration. The uncertainty is smaller than the symbol size.

The temperature dependence of the resistivity at different compositions is displayed in Figure 15. This isotherm plot shows a linear correlation between the absolute resistivity of the melt and the Bi concentration. The smooth dependence suggests that no obvious transitions are taking place in the melt with increasing Bi concentration. Furthermore, as the Bi concentration is increased, the slope of the resistivity curve increases as indicated from the distance between the isotherms. 

**Figure 16:**
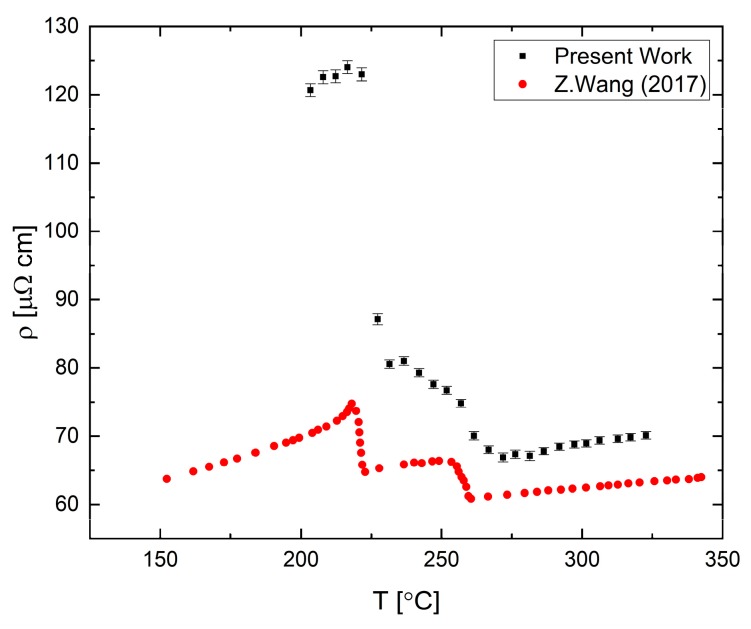
Resistivity of Bi_33_Ga_67_ liquid alloy compared to data from [40].

A measurement of the electrical resistivity of liquid Bi_33_Ga_67_ (in at.%) alloy near the melting point obtained during slow cooling with a rate of 60 °C/h is presented in Figure 16. The resistivity-temperature curve presents an abrupt change at 260 °C, about 50 °C above the liquidus. This change is correlated with phase separation in the melt [40]. Following this shoulder, a drastic increase of resistivity is observed below 225 °C, which is characteristic of the two-phase zone that contains a mixture of liquid and solid states. The difference between the present results and the results reported by Wang et al. [40] may originate due to use of DC vs. AC measurements which may produce an out of phase signal near the solidification or due to degradation of the contacts. 

**Figure 17:**
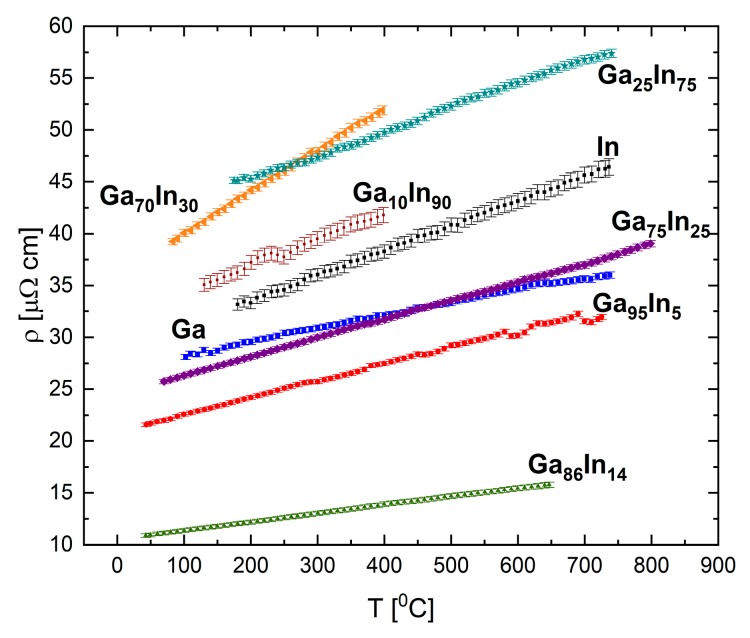
Electrical resistivity in the liquid Ga-In system as a function of temperature at selected alloy compositions. The uncertainty is smaller than the symbol size.

The resistivity of pure indium, gallium, and the selected binary alloys Ga_86_In_14_, Ga_70_In_30_, Ga_25_In_75_, and Ga_10_In_90_ (in at.%) were measured, and in Figure 17 we summarize the results for these compositions. The results present linear dependence of the resistivity with respect to temperature. No relation between the composition and the absolute resistivity values was found, in contrast to the Bi-Ga system. Two compositions exhibited the following outlying behaviors: the eutectic composition, Ga_86_In_14_, presented the lowest resistivity of the measured compositions; and the Ga_70_In_30_ had a significantly higher value of the resistivity-temperature slope. 

**Figure 18:**
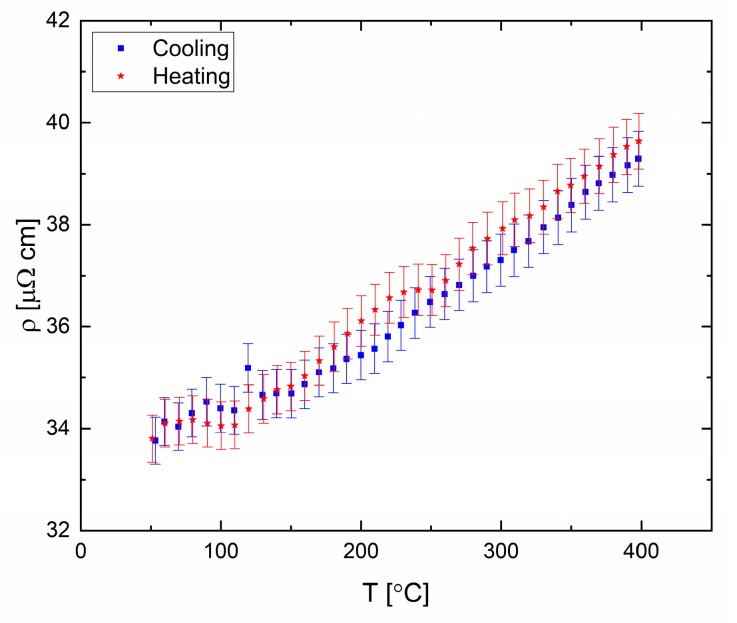
Electrical resistivity of eutectic Ga-In. Cooling and heating rate of 60 °C/h.

The eutectic alloy (Figure 18) displays abnormal behavior at 90 °C and 250 °C, which might indicate a possible transformation in the liquid phase. Structural transformation at 90 °C is seen upon heating and cooling cycles, which suggest a reversible process. A transformation at similar temperatures was reported previously based on XRD measurements [41]. 

**Figure 19:**
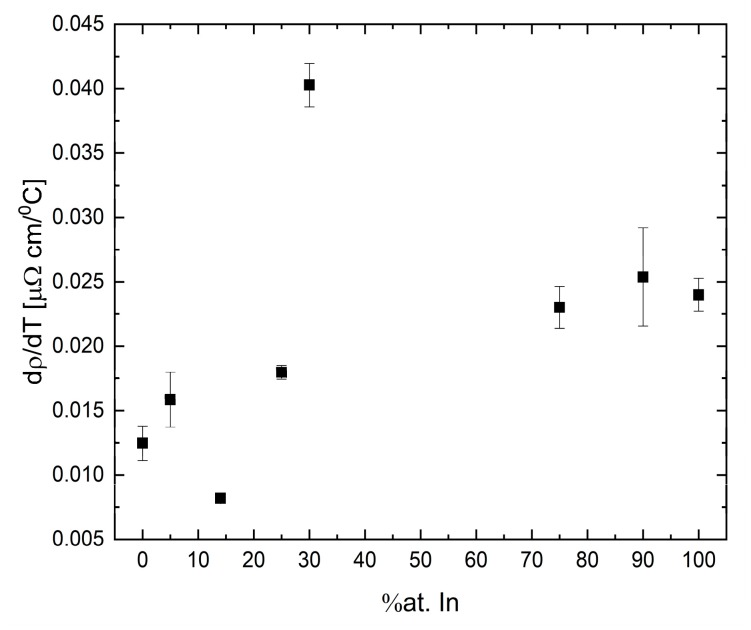
Resistivity coefficient of Ga-In as a function of indium concentration. Bars represent the uncertainty (symbols without bars have an uncertainty smaller than the symbol size).

The temperature coefficient of the Ga-In system presents no clear tendency with indium concentration, as can be seen in Figure 19. This result is in contradiction to the theory [42] for alloys with an equivalent amount of valence electrons, in which the change in composition will maintain the electron density unchanged and the trend should be linear.

**Figure 20:**
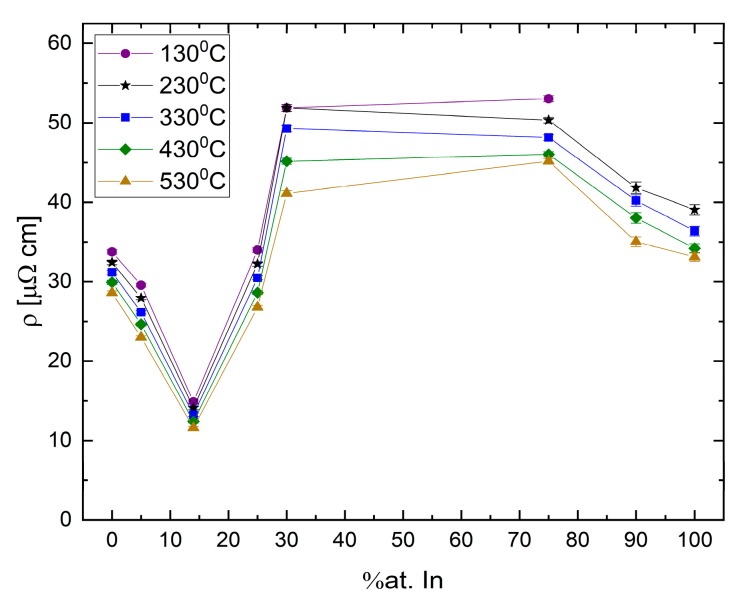
Absolute resistivity vs. indium concentration in Ga-In alloys.

The resistivity vallues as a function of composition for Ga-In binary alloys at several temperatures are presented in Figure 20. A unique phenomenon can be observed in these data, namely, that the hypereutectic area displays a general parabolic trend with a maximum resistivity between Ga_70_In_30_ and Ga_25_In_75_, and the hypoeutectic region shows a linear trend where the resistivity decreases until reaching a minimum at eutectic concentration, significantly below the resistivity of either elemental component.

## 6. Discussion

The present contribution presents an overview of experimental measurements and theoretical treatment to obtain physical and thermophysical properties of liquid metals and liquid binary alloys in the context of phase diagrams. We have carried out high-precision sound velocity, electrical resistivity and density measurements on several column III to V elements and their binary alloys. 

Measurements were carried out as a function of temperature in the liquid state. We found that, where relevant, the present measurements stand in a good agreement with previously published data. These measurements were used to map phase transformations by direct measurements, i.e., electrical resistivity, or by incorporating sound velocity and density measurements into a thermodynamic model to calculate phase diagrams of binary alloys under pressure. 

The measurements of the physical properties were also analyzed with respect to composition. Regarding sound velocity measurements, we observed that as the concentration of the element having a larger sound velocity is increased, the alloy sound velocity increases, respectively. Moreover, there is a connection between the elements’ temperature coefficient and that of the alloys. For example, if the two elements have normal behavior, then the alloys of both the elements present normal behavior as well. In the Pb-Sn system, both the elements and the different compositions of the alloy display normal temperature coefficients. In the Bi-Sb binary system, the Sb rich alloy displays anomalous behavior similar to Sb. As the Bi concentration increases, the behavior becomes semi-normal similar to in Bi. With regard to electrical resistivity, we observe that the Bi-Ga system shows a parabolic correlation with composition, as expected from mixed valence alloys. The Ga-In system, in which the elements have the same valences, presents a complex dependence on composition and not a linear ratio as expected [42]. 

Electrical resistivity is sensitive to structural changes, for example, as manifested upon a transition from a solid to liquid phase where the resistivity in metals usually increases. Furthermore, the thermal coefficient of the resistivity is also strongly connected to the structural properties, and thus a change in the slope can indicate a change in the liquid’s short-range order [32]. Such a change in the thermal coefficient is seen in the Bi-Ga system and is related to the region of the phase diagram presenting a phase separation in the liquid. The electrical resistivity measurements indicate, with high accuracy, these phase transformations from a homogenous liquid to the two liquids region and, then, to the zone of mixed phases of liquid and solid. 

The use of CALPHAD allows us to exploit data measured at ambient pressure to calculate phase diagrams of binary alloys under pressure. The sound velocity and the density are used to calculate the pressure dependence of the interaction parameters on pressure to the second order. We apply this formalism for two examples, the isomorphic system Bi-Sb and the eutectic system Pb-Sn. 

We demonstrated the use of experimental measurements to obtain nontraditional thermal analysis. These are thermophysical measurements that can shed some light on the dependence of the structure on temperature, and provide evidence on the presence or absence of a local change in the liquid state. From sound velocity and density measurements at ambient conditions, we are able to calculate the pressure dependence of the interaction parameters in the liquid state, which is required to calculate the phase diagram under pressure of binary systems. This provides an innovative path to predict properties of the material under pressure and the dependence of eutectic point and liquidus temperature as a function of composition. 

## Supplementary Materials

The following are available online at https://www.mdpi.com/1996-1944/12/23/3999/s1.
